# American Medicinal Plants

**Published:** 1884-10

**Authors:** 


AMERICAN MEDICINAL PLANTS: An Illustrative and Descriptive Guide, etc. By C. F. Millspaugh, M. D. First fascicle. Boericke & Tafel: New York and Philadelphia.

We can thoroughly congratulate Dr. Millspaugh on the beautiful and accurate plates he has given of these plants. Nothing more life-like can be found, and the accompanying descriptive text is brief and accurate, the whole making a work which all should possess and be proud of.

The work is to be issued in fascicles of five parts, each containing *thirty* plates, at five dollars per fascicle. The publishers hope to complete the work in two years: *now* is the time to subscribe.

